# Review of: Appalachian Health: Culture, Challenges, and Capacity

**DOI:** 10.13023/jah.0501.08

**Published:** 2023-04-01

**Authors:** Danielle M. Davidov

**Affiliations:** West Virginia University, ddavidov@hsc.wvu.edu

**Keywords:** Appalachia, Appalachian history, book review, geography, health disparities, public health

## Abstract

The *Journal of Appalachian Health* is committed to reviewing published media that relates to contemporary concepts affecting the health of Appalachia. This is a review of the text *Appalachian Health: Culture, Challenges, and Capacity.* This book is an ideal starting point for anyone interested in Appalachian history, public health, and health disparities research.

## MEDIA TYPE: BOOK

### CITATION

Scutchfield FD, Wykoff RW, editors. Appalachian health: culture, challenges, and capacity. Lexington KY: University Press of Kentucky, 2022. ISBN-13: 978-0813155576; Cost: $40.50 e-Edition; $45.00 Hardcover.

### ABOUT THE REVIEWER

**Danielle M. Davidov, PhD**, is an associate professor in the Schools of Public Health and Medicine at West Virginia University. Her research employs qualitative and mixed methods to develop and evaluate complex interventions to address trauma and violence in community-based, academic, and clinical settings. Much of her work has focused on the intersection of trauma, substance abuse, and mental health among Appalachian populations. She has received grant funding from the National Institute of Minority Health and Health Disparities, Canadian Institutes of Health Research, and Patient-Centered Outcomes Research Institute to explore and address issues related to intimate partner violence and child maltreatment in Appalachia. She conducts research, teaching, service, and engagement activities that aim to enhance the health and well-being of women and underserved groups in her home state of West Virginia. Within the School of Public Health, she mentors undergraduate, master’s-level, and doctoral students and teaches courses related to intervention design, qualitative methods, social determinants of health, and public health prevention.

### ABOUT THE AUTHOR

This book is comprised of 11 chapters written by scholars and researchers from public health disciplines (e.g., epidemiology and biostatistics, health services research, health equity, rural health, addiction science, health policy, and environmental and occupational health) as well as experts in the field of Appalachian studies. Many of the authors are natives of Appalachia and currently live and work within the region.

Editors include F. Douglas Scutchfield, MD, and Dr. Randy Wykoff, MD, MPH, TM. Dr. Scutchfield, a renowned public health expert and leader in the field of Appalachian health, passed away in May 2022. Dr. Scutchfield held appointments in the Honors College, College of Medicine, and College of Public Health at the University of Kentucky (UK). He founded the College of Community Health Science at the University of Alabama and the Graduate School of Public Health at San Diego State University. He additionally founded the School (now College) of Public Health at UK. In addition to extensive editing and publishing experience in the field of public health, he served as the founder and previous editor-in-chief of this journal.

Dr. Randy Wykoff is the current editor-in-chief of the *Journal of Appalachian Health* and co-editor of the book under review. He is Founding Dean of the College of Public Health and Director of the Center for Rural Health Research, both at East Tennessee State University. He is board certified in Pediatrics and Preventive Medicine, with an additional certification in Tropical Medicine. Previously, he served as Senior Vice President for International Operations at Project HOPE, as the Deputy Assistant Secretary for Health (Disease Prevention and Health Promotion) in the U.S. Department of Health and Human Services, and for 11 years with the Food and Drug Administration.

### THE REVIEW

Aunique region with a complicated social, political, and economic history, Appalachia often enters the public discourse as a place bound by geography and culture that is subjected to stereotypes and fraught with health challenges. Appalachia has long been mistaken as an area of abject poverty begging for outside intervention. Still, the region faces significant socioeconomic and health disparities that threaten the health and well-being of current and future generations. A critical first step in reducing these disparities is to document them, examine their causes, and propose evidence-based, multilevel solutions.

This text uses a socio-ecological approach to provide an in-depth examination of the complex history and current reality of health in the U.S. Appalachian Region. It takes the reader on a fascinating journey that illustrates how the mountainous region, with one of the nation’s strongest economies prior to the Civil War, and an abundance of natural resources, became home to some of the most distressed and disadvantaged places in the country within just over a century. The authors do an impressive job of describing the region and its inhabitants not as a monolith, but in a way that highlights challenges as well as bright spots using historical insights into the causes and context surrounding Appalachian health.

This book is an ideal starting point for anyone interested in Appalachian history, public health, and health disparities research. More established scholars (e.g., authors, teachers, and researchers) and their students who focus on Appalachian health will appreciate the myriad comparisons between Appalachian and non-Appalachian counties on a variety of health indicators. Additionally, the inclusion of data demonstrating the geographic variation in health and economic status within Appalachian subregions can help planners, politicians, and researchers focus energy and resources on places most in need. Epidemiological data provided throughout the text reveal that even where improvements in Appalachian health and socioeconomic status have been made, this progress has happened at a much slower pace than in the rest of the U.S.

To explain and provide context for these disparities, the authors expertly weave together consequential stories of the region’s rich and complicated history, including hardships like environmental exploitation and mining disasters, as well as powerful success stories, such as the formation of the Frontier Nursing Service and Appalachian Regional Healthcare System. Importantly, this text emphasizes the complex interplay of place, identity, and cultural context, while underscoring the substantial power and influence that structural and political forces have had on Appalachia. Taken together, the chapters of this book provide “a cautionary tale but inspiring fable” of a region of paradox and tremendous promise. Alongside the epidemiological data are stories about local communities and champion changemakers that give us hope for the future with inspiration from the past.

This book contains numerous tables, graphs, and figures illustrating comparisons of sociodemographic and health indicators between non-Appalachian counties, Appalachian counties, and in many cases between subregions of Appalachia (Central, Northern, Southern, etc.). There are also tables showing Appalachian and non-Appalachian metrics of healthcare utilization and access as well as data on trends over time for numerous variables and indicators. Included maps show county-level health disparities and indicators throughout the Appalachian Region. To top these resources off, there are extensive endnotes and references at the end of every chapter.

#### Relevance to Appalachian Health

This book is an in-depth, nuanced exploration of health in Appalachia, with focus on disparities and health indicators described within relevant geographical, historical, socio-cultural, and structural contexts. While the reader is left with the stark reality that Appalachians unquestionably suffer poorer health and quality of life compared to their non-Appalachian counterparts, we are also reminded of the preventable, reversible, and thus changeable nature of many of the outlined health indicators. It provides us with reassurance that while we have a long way to go to make meaningful improvements in Appalachian health, with solution-oriented, structural, and environmental approaches, the issues are surmountable.

Finally, the authors leave us with a charge: Appalachian problems are the world’s problems. If we can learn from the complex lived experiences of Appalachian people to find equitable solutions to their health challenges, those same solutions can be applied globally and locally to improve health.

